# Repeatability of the “flash-replenishment” method in contrast-enhanced ultrasound for the quantitative assessment of hepatic microvascular perfusion

**DOI:** 10.1590/1414-431X20177058

**Published:** 2018-02-26

**Authors:** Fang Xie, Wen-Bo Wan, Xiang Fei, Ming-Bo Zhang, Yan Zhang, Hong-Wei Wang, Jie Tang, Wen-Bo Tang, Yu-Kun Luo

**Affiliations:** Department of Ultrasound, Chinese PLA General Hospital, Beijing, China

**Keywords:** Flash-replenishment, Contrast-enhanced ultrasound, Liver ischemia-reperfusion injury, Microvascular perfusion, Rabbits

## Abstract

This study aimed to evaluate the feasibility and repeatability of the flash-replenishment method in contrast-enhanced ultrasound (CEUS) perfusion imaging and assess quantitatively microvascular perfusion in the liver. Twenty healthy New Zealand rabbits were submitted to CEUS perfusion imaging with continuous intravenous infusion. Using flash-replenishment kinetics, the dynamic process of depletion and refilling of microbubble contrast agent was recorded. The hepatic microvascular perfusion parameters were calculated, including region of interest, peak intensity (PI), area under the curve (AUC), and hepatic artery to vein transit time (HA-HVTT). A consistency test was performed for multiple measurements by the same operator and blind measurements by two different operators. The hepatic perfusion imaging of 3×10^8^ bubbles/min had minimal error and the best imaging effect and repeatability. The variability of the perfusion parameter measured at 3 cm depth under the liver capsule was at a minimum with coefficient of variation of 3.9%. The interclass correlation coefficient (ICC) of measurements taken by the same operator was 0.985, (95% confidence interval, CI=0.927-0.998). Measurements taken by two operators had good consistency and reliability, with the ICC of 0.948 (95%CI=0.853-0.982). The PI and AUC of liver parenchyma after reperfusion were lower than before blocking; and HA-HVTT was significantly longer than before blocking (P<0.05). The flash-replenishment method in CEUS perfusion imaging showed good stability and repeatability, which provide a valuable experimental basis for the quantitative assessment of hepatic microvascular perfusion in clinical practice.

## Introduction

Contrast-enhanced ultrasound (CEUS) perfusion imaging is a noninvasive functional imaging used to assess different pathophysiological and anatomical structures associated with disease through the study of blood flow, metabolism, and changes in the receptors on specific tissues and organs (1–3). The ultrasonic contrast agent containing microbubbles was delivered to tissues and organs through intravenous injection. These microbubbles can serve as indicators, helping clinicians monitor changes in microbubble concentration at the same position over time to assess the time-intensity curve (time as x-axis and intensity as y-axis), which can be used to measure the perfusion parameters in different tissues and organs through different injection methods and the fitting of different mathematical models.

Microcirculation, a basic unit of liver structure and function, affects liver function and the degree of hepatic injury to a considerable extent (4). Sufficient perfusion allows the maintenance of biological activity and functions of liver tissue, as changes of tissue and organ perfusion are detectable in the early stages of most diseases. The evaluation of hepatic microvascular perfusion provides important information regarding the early stages of specific diseases. The use of microbubble contrast agent as vascular tracer and enhancer allows the functional imaging of hepatic microvascular perfusion before morphological changes of tissues can be detected, which is advantageous compared to traditional ultrasound imaging (5–7).

CEUS perfusion imaging was originally and primarily used for the evaluation of myocardial perfusion (8). A number of studies have attempted to use contrast-enhanced quantitative analysis curves to evaluate the perfusion of brain tissues (9) and kidneys (10–12), the degree of regenerative blood vessels in tumors (2), and the grade of liver cirrhosis (13), suggesting the evaluation has good application prospects.

Color Doppler imaging has been the mainstream for the evaluation of liver hemodynamics. The recent CEUS perfusion imaging of the liver is primarily performed with contrast administered via intravenous bolus injection. However, the rapid contrast clearance greatly decreases the stability of contrast enhancement and the accuracy of hemodynamics evaluation (14). The unstable contrast concentration in tissues has been a major problem in blood perfusion evaluation of CEUS. Continuous infusion of contrast with flash-replenishment kinetics can achieve a stable contrast concentration in tissues (15). Upon this condition, the contrast infusion rate is correlated with the local blood flow velocity, and the microbubble amount is correlated with the microvascular blood volume. Therefore, continuous infusion of contrast can be a useful tool in evaluating organ blood perfusion.

## Material and Methods

### Preparation of experimental animals

Twenty healthy male and female New Zealand rabbits weighing 3.0-3.5 kg were provided by the Chinese PLA General Hospital Experimental Animal Center [Experimental animal license number: SCXK (Beijing) 2010-0001]. The research protocol for this study was approved by the Experimental Animal Welfare Ethical Review Committee of the Chinese PLA General Hospital (2015-x10-11). All rabbits were housed in the thermostat-controlled animal facility with free access to food and water for 2 adaptive weeks, and none showed any abnormality. Animals were fasted for 12 h with free access to water before CEUS perfusion imaging.

### CEUS perfusion imaging

#### Contrast agent

SonoVue contrast agent was purchased from Bracco (Italy). Membrane materials used in this study consisted of polyethylene glycol and phospholipids and filled with stable sulfur hexafluoride (SF_6_) gas. It was mixed with 5 mL normal saline to form a white, milky microbubble suspension before use. Each microliter of microbubble suspension contained 8 µL/mL (1-5×10^8^ bubbles/mL, average of 3×10^8^ bubbles/mL) SF_6_.

#### CEUS perfusion imaging procedures

All conventional ultrasonography and CEUS perfusion imaging were conducted using iU Elite Extreme color ultrasound diagnostic system (Philips Medical Systems, USA) and C5-1 abdominal probe for imaging at 3-5 MHz frequency. Transverse sections of the right hepatic lobe were the target area of observation and the image was locally amplified to enter contrast side/side dual imaging mode. To ensure imaging quality, the calibration parameters of the diagnostic system were defaulted to the same standard: 0.05 mechanical index (MI), 64% time gain compensation, 5.0 cm imaging depth, and 18 Hz frame rate. The gain adjustment was set so that the two-dimensional grayscale background echo was barely visible, and the focus was placed on the distal end of the observed target area (the observed target remained unchanged in the two imaging examinations before and after modeling), which remained unchanged throughout the imaging procedures. The interval between two injections was at least 30 min to ensure complete removal of the microbubbles from the blood microcirculation.

SonoVue contrast agent was continuously infused using an intravenous micro-dosage pump at a slow and constant speed until the end of the experiment. The infusion liquid was constantly shaken during the infusion to produce an evenly mixed suspension. As the microbubbles were continuously infused and the concentrations became stable, "flash-replenishment" was used with the probe fixed at a stabilized position. We then selected the contrast timer, capture, and flash modes, using high mechanical index pulse (MI 1.20) to destroy the microbubbles in the ultrasound irradiation area, and immediately switched to the low mechanical index pulse imaging mode. The dynamics of the disappearing-refilling process were continuously monitored and stored in DICOM format, including the following three stages: the steady state before the destruction of the contrast agent, the instantly enhanced destruction of microbubbles after flash destruction, and the complete examination of microbubble contrast agent reperfusion of the tissues adjacent to the transverse sections (until the microbubbles again reached the fully enhanced steady state).

### Image processing and analysis

After CEUS perfusion imaging, the ultrasound images were processed using the QLAB software (Philips Medical Systems). The exponential function I(t)=A(1−e^−βt^)+C was used for the time-intensity curve fitting of the reperfusion in the region of interest (ROI), with the area of ROI at 25.62 mm^2^. The time-intensity curve of the tissue reperfusion ROI was used to measure the hepatic microvascular perfusion parameters. In the function, A represents the intensity of the plateau, i.e., the maximum amplitude of the reperfusion curve, reflecting the peak intensity (PI) of perfusion; β represents the rate, i.e., the slope of the reperfusion curve, reflecting the blood flow rate; and AUC represents the area under the curve, indicating the tissue blood volume of perfusion.

Hepatic artery to vein transit time (HA-HVTT) represents the difference of arrival times of microbubbles first observed in the ultrasonography between the right branch of hepatic artery and the right branch of hepatic vein.

In this study, image analysis was performed in a blind fashion by two physicians with 10 years of diagnostic experience in abdominal ultrasound and 1 year of CEUS experience. The physicians did not know the experimental grouping. The inconsistent results were reanalyzed blindly by the third senior physician. Each set of data was measured three times to find the mean of the measurement data from the two physicians, which served as the final measurement value.

### Stability and repeatability of the perfusion parameters

#### Adjustment of perfusion rate of the contrast agent

To obtain the best images of hepatic microvascular perfusion, the impact of different perfusion rates on the hepatic microvascular perfusion parameters were observed. Twenty healthy New Zealand rabbits were anesthetized with intramuscular injection of 0.1-0.2 mL/kg Sumianxin, and infused slowly with the contrast agent using a continuous micro-dosage pump with the perfusion rates of the contrast agent adjusted as 2×10^8^, 3×10^8^, and 4×10^8^ bubbles/min. After CEUS perfusion imaging, PI was measured to evaluate the perfusion rate of the contrast agent during the optimal hepatic microvascular perfusion imaging.

#### Repeatability of perfusion parameters

To determine whether the selection of ROI affected perfusion parameters and evaluate the stability and repeatability of the parameters in CEUS perfusion imaging, we selected 20 healthy New Zealand rabbits to measure the PI of ROI at different depths (2, 3, and 4 cm from the hepatic capsule) and the PI of different ROI positions (positions a and b) at the same depth.

#### Measurement of consistency between operators

The interclass correlation coefficient (ICC) is one of the indicators used to evaluate the reliability and repeatability of measures performed by different operators. In general, ICC >0.75 indicates good reliability, so higher ICC values are often required for quantitative data.

The consistency test was performed using the ICC of the blind measurement results (i.e., PI) collected from the multiple measurements performed by the same operator and by two operators under the same measurement conditions.

### Quantitative evaluation of the hepatic microvascular perfusion features after ischemia/reperfusion injury

**Figure 1.**
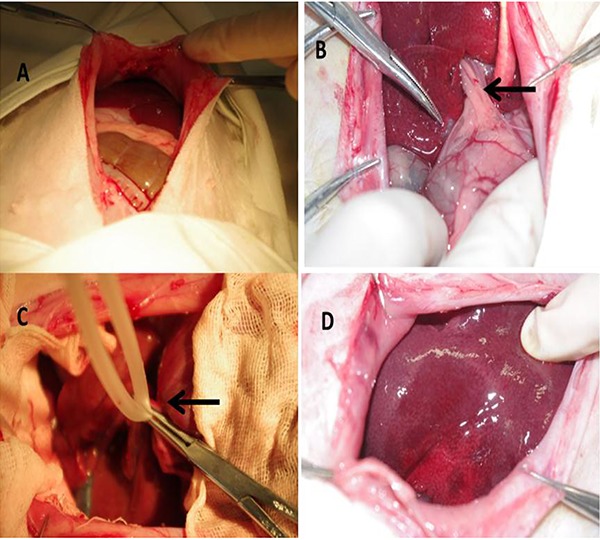
Pringle's maneuver. A 6-cm midline incision was carried out (*A*) and the porta hepatis was exposed (*B*). The hepatic artery proper, common hepatic duct and hepatic portal vein were blocked using three vascular clips for 60 min (*C*) followed by 120-min reperfusion (*D*).

Twenty rabbits were anesthetized with intravenous injection of 3% pentobarbital sodium (30 mg/kg). The Pringle's maneuver was used to induce *in vivo* liver ischemia/reperfusion (I/R) injury (16). The rabbit was put into the supine position and the abdomen skin was shaved and disinfected with 0.5% iodophor. A 6-cm midline incision was carried out (Figure 1A) and the porta hepatis was exposed (Figure 1B). The hepatic artery proper, common hepatic duct and hepatic portal vein were blocked using three vascular clips for 60 min (Figure 1C) followed by 120-min reperfusion (Figure 1D). The liver color shifting from dark red to bright red in 2-5 min after releasing the clip indicated successful establishment of the I/R injury model. All animals were subjected to CEUS perfusion imaging using the aforementioned methods before blocking and after reperfusion to assess the hepatic microvascular perfusion parameters, i.e., PI, AUC, and HA-HVTT.

### Statistical analysis

SPSS 24.0 (SPSS Inc., USA) software was used for the statistical analysis of this study. P<0.05 was considered a statistically significant difference. Data are reported as means±SD. One-way ANOVA for completely randomized design was used to perform comparisons across multiple groups, followed by student-Newman-Keuls (SNK)-q *post hoc* test. The variabilities were calculated to evaluate the stability of the contrast agent parameters. ICC was used to measure the repeatability, and the independent samples *t*-test was used to compare the mean of two groups.

## Results

### Adjustment test for perfusion rate of contrast agent

**Figure 2.**
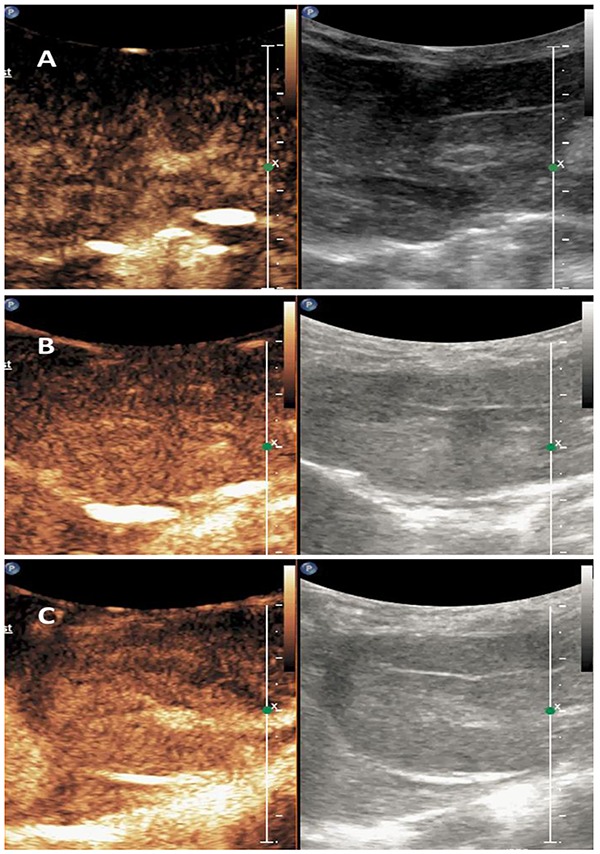
Hepatic perfusion imaging of different contrast agent perfusion rates. The 3×10^8^ bubbles/min (*B*) and 4×10^8^ bubbles/min (*C*) perfusion rates showed better enhancement than 2×10^8^ bubbles/min (*A*). Additional tests showed that the 3×10^8^ bubbles/min perfusion rate was superior to the 4×10^8^ bubbles/min perfusion rate in image enhancement and repeatability.

Different perfusion rates, i.e., 2×10^8^, 3×10^8^, and 4×10^8^ bubbles/min were independently used to slowly and continuously infuse the contrast agent in CEUS perfusion imaging. The PI of the optimal hepatic perfusion imaging effect in the 20 rabbits under different perfusion rates were 13.28±0.69, 16.33±0.36, and 16.72±0.90 dB, respectively (P<0.05, Figure 2).

**Figure 3.**
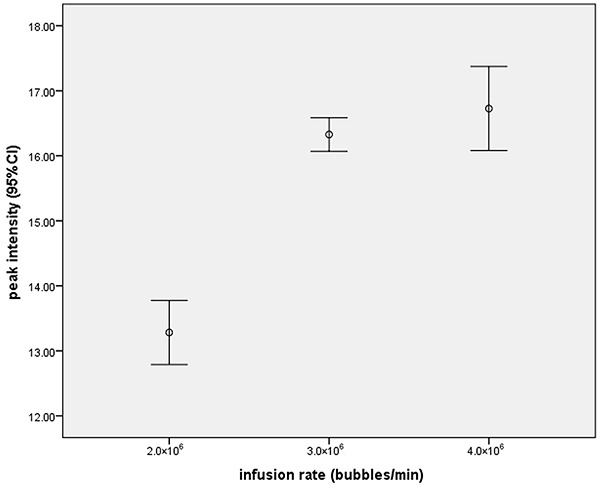
Hepatic perfusion parameter (peak intensity) error bars of different contrast agent perfusion rates. Each bar represents the average 95%CI.

After SNK-q *post hoc* testing, 3×10^8^ and 4×10^8^ bubbles/min perfusion rates had the same imaging effect (P>0.05), which was better than the 2×10^8^ bubbles/min perfusion rate (P<0.05). However, as shown in Figure 3, the hepatic PI (95%CI) error bar of the group with a perfusion rate of 3×10^8^ bubbles/min was the lowest, with the best repeatability and the most stable imaging effect. For this reason, to achieve the best hepatic perfusion imaging effect, we selected 3×10^8^ bubbles/min for infusion of the contrast agent in later experiments.

### Stability of perfusion parameters of the contrast agent

The CEUS perfusion imaging results showed that the differences were significant in PI values at depths of 2, 3, and 4 cm from the liver capsule in the ROI, with coefficients of variation (CV) of 30.3, 3.9, and 13.0%, respectively (P<0.05, Table 1). The results showed that the PI value at 3 cm depth from liver capsule had the least variability while the errors of PI measured at depths of 2 and 4 cm from the liver capsule were larger.

**Table 1.** Comparison of peak intensity (PI) at different depths beneath the liver capsule.

Depth	PI (dB)	*F*	P
2 cm	3.36±1.02	66.037	<0.001
3 cm	9.15±0.36* #		
4 cm	6.49±0.86		

Data are reported as means±SD.

* P<0.001, relative to the 2 cm depth from the liver capsule;

# P<0.001, relative to the 4 cm depth from the liver capsule (ANOVA).

**Figure 4.**
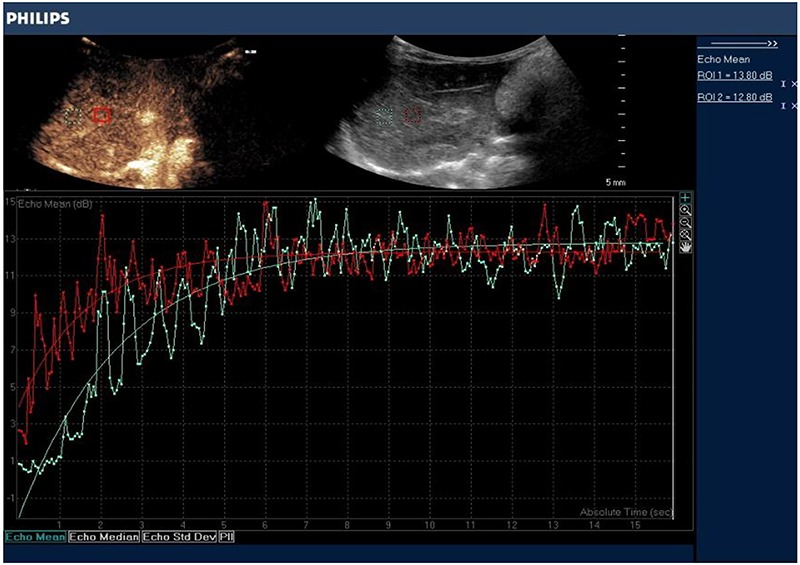
Hepatic perfusion time-intensity curves at different regions of interest positions at a depth of 3 cm from the liver capsule. The green square and the green curve denote position a. The red square and the red curve denote position b. The two positions were not significantly different in peak intensity (P=0.266, *t*-test).

PI values at depths of 3 cm from the liver capsule in different ROI positions (positions a and b, Figure 4) showed 11.9 and 8.0% CV, respectively, but the differences were not significant (P=0.266, Table 2).

**Table 2.** Comparison of peak intensity (PI) at different regions of interest positions at a depth of 3 cm from the liver capsule.

Position	PI (dB)	*t*	P
a	8.84±1.06	1.214	0.266
b	8.16±0.66		

Data are reported as means±SD. Student’s *t*-test was used for statistical analysis.

The depth from the liver capsule had more significant impact on the perfusion parameters than the horizontal positions of the ROI. For this study, we chose the depth of 3 cm from the liver capsule to measure the perfusion parameter in ROI.

### Consistency of operators

#### Repeatability of the same operator

A single operator selected randomly 20 healthy New Zealand rabbits for CEUS perfusion imaging on the same day to measure the PI of perfusion parameters. The ICC of the 20 measurements was 0.985 (95%confidence interval, CI=0.927-0.998), which indicate good repeatability and small measurement error.

#### Consistency and reliability of two different operators

Two operators selected randomly 20 healthy New Zealand rabbits for CEUS perfusion imaging under the same conditions to measure the PI of perfusion parameters in a blind fashion. The ICC of measurements was 0.948 (95%CI=0.853-0.982), suggesting consistency and good reliability.

### Hepatic microvascular perfusion features after I/R injury

**Figure 5.**
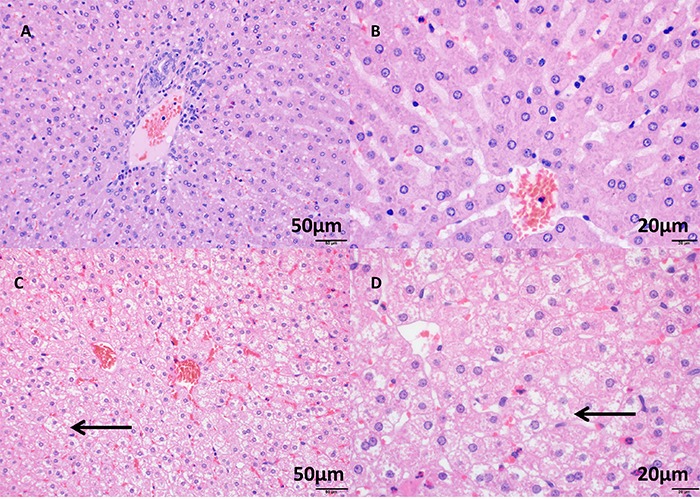
HE staining of liver tissue. The normal hepatocytes are well arranged with centered nuclei, sharp nuclear membrane and red-stained cytoplasm (*A*, *B*). After the ischemic/reperfusion injury, the hepatocytes are swollen and the hepatic cords are disorganized (arrow in *C*). The cytoplasm is lightly stained with vacuoles (arrow in *D*).

**Figure 6.**
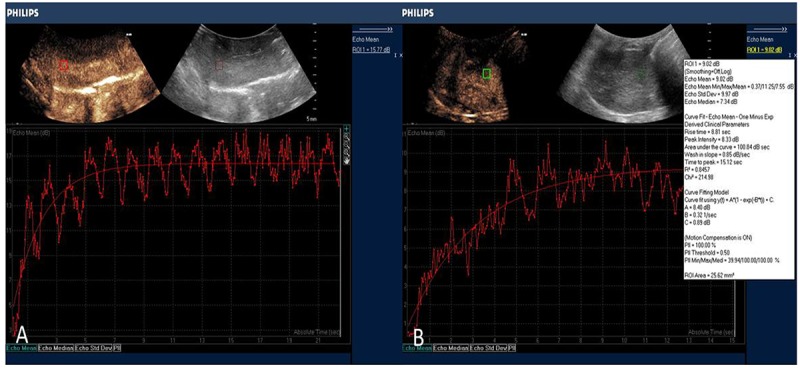
Hepatic perfusion time-intensity curves before blocking (*A*) and after reperfusion 120 min (*B*).

The liver I/R injury model was successfully established in 20 healthy New Zealand rabbits and was verified histologically (Figure 5). The time-intensity curves showed that the PI and AUC of liver microvascular perfusion after reperfusion were lower than before blocking (P<0.05). The HA-HVTT of liver parenchyma after reperfusion was longer than before blocking (P<0.05, Figure 6, Table 3).

**Table 3.** Comparison of hepatic perfusion parameters before blocking and after reperfusion.

Group	PI (db)	AUC (db s)	HA-HVTT (s)
Before blocking	16.82±1.69	305.37±33.67	15.42±1.04
After reperfusion	8.03±0.52	96.99±11.90	23.56±1.78
*t*	15.734	18.452	12.493
P	<0.001	<0.001	<0.001

Data are reported as means±SD. Student’s *t*-test was used for statistical analysis. PI: peak intensity; AUC: area under the curve; HA-HVTT: hepatic artery to vein transit time.

## Discussion

CEUS perfusion imaging is a functional imaging modality. The contrast used in our study was sulphur hexafluoride microbubbles with a mean diameter of 2.5 μm. The size of microbubbles is similar to erythrocytes and has no obvious disturbance to hemodynamics. The theoretical basis for its use in the evaluation of organ perfusion is that microbubbles used in contrast-enhanced ultrasonography can generate good harmonic imaging, and have a strong scattering effect on ultrasound. Thus, the system detects blood flow down to approximately 1 mm/s in the capillaries, which is a significant improvement in the sensitivity of ultrasound with respect to detecting low-speed blood flow, thereby facilitating ultrasonic evaluation of the microvascular perfusion of the tissue.

In most published studies, the contrast agent was administered using intravenous bolus injection. The unstable contrast concentration in tissues has been a major problem in blood perfusion evaluation of CEUS. Continuous infusion of contrast with flash-replenishment kinetics can achieve a stable contrast concentration in tissues. Upon this condition, the contrast infusion rate is correlated with the local blood flow velocity, and the microbubble amount is correlated with the microvascular blood volume. We first measured the stability and repeatability of the perfusion parameters of CEUS perfusion imaging and then assessed quantitatively the hepatic microcirculatory function using a variety of mathematical function models by applying harmonic imaging techniques, intermittent power Doppler imaging, and grayscale CEUS.

Our study found that ROIs with the same transverse position but different depths yielded significantly different PI values. On the contrary, ROIs with the same depth but different transverse positions were not significantly different concerning PI. Near the acoustic beam, the microbubbles receive more energy and thus are more frequently disrupted, resulting in attenuated contrast enhancement. In addition, the acoustic energy decreases with the increased depth, and the acoustic beam is heterogeneous in different imaging planes, both of which result in enhanced attenuation. Therefore, the ROI depth but not its transverse position determines the PI value. In our study, all the hemodynamics parameters were measured in ROIs with a 3-cm depth.

To minimize the differences in perfusion parameter measurement, we adjusted the perfusion rates (doses) of the ultrasonic contrast agent to achieve a more accurate quantitative assessment of the perfusion features. The optimum perfusion rate was defined as 3.0×10^8^ bubbles/min, which showed superior results in image enhancement and repeatability.

Liver perfusion imaging has been increasingly used in studies of liver tumors and cirrhosis. Many previous studies used a relatively small number of experimental outcomes (17–19). Our study showed that liver I/R injury causes a series of changes including microcirculation dysfunction, reduction in blood flow volume and velocity in the hepatic sinus, decreased PI and AUC and prolonged HA-HVTT. Our results suggest PI, AUC and HA-HVTT can be used to accurately and quantitatively assess blood flow velocity and volume, and the time of hepatic microvascular perfusion, and thus effectively evaluate the hepatic microcirculatory dysfunction of I/R injury.

CEUS perfusion imaging had advantages that cannot be found in other imaging examinations. The method required lower doses of contrast agent, and had no toxic side effects. It also provided real-time imaging and dynamic observation at a very low commercial price. CEUS was highly suitable for the study of tissue microvascular perfusion. With further improvement and development of CEUS technology and quantitative analysis software, this methodology can be highly efficient for the assessment of tissue microvascular perfusion, and become a suitable option for clinical practice.
